# Investigation of dry sliding wear and mechanical properties of hybrid epoxy composites reinforced with pineapple leaf and roselle fibers

**DOI:** 10.1038/s41598-025-10431-1

**Published:** 2025-07-31

**Authors:** K. Giridharan, B. Elumalai, M. Vinothkumar, G. Chakravarthi, D. Manikandan, Krishnaraj Ramaswamy

**Affiliations:** 1 Department of Mechanical Engineering, Easwari Engineering College, Chennai, Tamilnadu India; 2 Department of Aeronautical Engineering, Vel Tech Rangarajan Dr. Sagunthala R&D Institute of Science and Technology, Chennai, Tamilnadu India; 3 Department of Mechanical Engineering, SRM TRP Engineering College, Tiruchirappalli, Tamilnadu India; 4 Department of Mechanical Engineering, College of Engineering and Technology, Dambi Dollo University, Dambi Dollo, Ethiopia; 5 Center for global health research, Saveetha institute of medical and technical sciences, Saveetha University, Chennai, Tamilnadu India

**Keywords:** Natural fiber, Roselle fiber, PALF, Hybrid composite, Mechanical properties, Wear, Engineering, Mechanical engineering

## Abstract

Natural fiber composites have gained significant potential in past decades due to their favorable physical properties and mechanical characteristics. Pineapple leaf fibers and roselle fibers are abundant agricultural wastes and tend to be recycled to minimize environmental pollution. The present work aims to investigate and analyze the effects of reinforcing pineapple leaf fiber (PALF) and roselle fiber with epoxy hybrid composites. The composites are fabricated using the hot compression molding process. The mechanical attributes of the composites are studied through tensile, flexural, and impact tests. Along with a hybrid combination of fibers, a neat resin sample and samples of PALF and roselle fiber alone were also fabricated for test comparison. The wear behaviour of the fabricated specimen was explored by pin-on-disc wear test and examined the specific wear rate and co-efficient of friction properties. The fractographic studies of the hybrid composite fractured specimen through tensile and impact tests and the worn surface from the wear tests were analyzed using a scanning electron microscope. The results discovered that 25 wt% of PALF with 25 wt% of roselle fiber composite showed significant improvements in tensile characteristics, flexural and impact strength of 42.03 MPa, 38.17 MPa, and 46 KJ/m^2^. The best wear resistance was observed for 30 wt% PALF with 20 wt% of roselle fiber hybrid composite. Based on the experiments, hybrid fiber composite sample getting better wear and mechanical properties and suggesting their suitability to applications for cabinets like wardrobes, cupboards, and storage shelves.

## Introduction

### Motivation and sustainability

In the past years, natural fiber composites have attracted more interest among researchers due to their desirable mechanical aspects, environmental friendliness, and cost-effective nature^1^. Recent studies on fibre composites^2^ reveal how natural fillers can improve mechanical properties and promote environmental sustainability in polymer composites. Polymer resins reinforced with such fibers offer comparable mechanical strength to synthetic fiber composites. Many agricultural by-products are remained unutilized and come under the category of “waste”^3,4^. This has motivated researchers to transform crop and food processing wastes and utilize them as suitable forms of fiber. The processing of agro waste into fibers requires less energy and promotes sustainable development.

### Individual fiber properties

Among the agro-wastes, PALF is derived from the pineapple plants leaves and it is recognized for its high cellulose content and favorable mechanical properties^5,6^. Additionally, roselle fiber is a sustainable natural fiber that exhibits good mechanical properties and favorable environmental benefits^7^. One of the most widely used thermosetting polymers in composite processing is epoxy resin matrix. It is collectively known for its adhesive properties, chemical resistance, and excellent mechanical behavior. Compared to other resin matrices, epoxy provides low shrinkage and proper dimensional stability during curing^8^. The major drawback of natural fibers is their hydrophilic nature, which results in reduced adhesive properties. This limitation was rectified by modifying the surface property through a chemical treatment process^9^.

### Epoxy resin role

Epoxy resin plays a crucial role in composite materials, especially when combined with natural fibers. It serves as the matrix that binds the fibers together, distributing loads effectively and enhancing structural integrity^10,11^. Epoxy is favored for its strong adhesion, good thermal stability, and resistance to environmental factors. When used with fibers like pineapple leaf fiber (PALF) and roselle fiber, it helps form a strong interface that is essential for mechanical performance^12^. The resin also helps minimize voids and ensures uniform dispersion of fibers, which is important for consistent strength and wear resistance in hybrid composites.

### Hybrid composite advantage

Hybrid fiber composites have emerged as a superior alternative to single fiber composites because of their combined ability of strength with tailored properties^13,14^. The benefits of hybrid reinforcement, such as improved mechanical and tribological properties, align with findings in laser-clad materials^15^ and polymer composites with hybrid fillers^16^. The single fiber composites are often limited by their inherent weakness of the properties. But the multiple fibers in hybridized composites to provide better improvements in mechanical, thermal, and tribological performance^17,18^. Natural fiber composites have found widespread applications across various manufacturing industries due to their excellent physical and mechanical properties^19^. The applications such as seat backs and interior panels have been developed to improve to improve the fuel efficiency in the automotive sector^20^. The development of wall panel, roofing sheets, and insulation materials with enhanced strength, corrosion resistance, and durability in construction zone^21^. Another significant sector, which was overwhelmed by natural fiber composites is furniture industries. The composites have been increasingly used in wardrobes, and shelves due to their cost-effective and light weight applications^22,23^. The major factor that supports the application of composites is to provide the sufficient wear resistance. The study of friction, wear, and lubrication is the suitable way to improve the tribological behaviour of the composites^24,25^. The hybridization of pineapple leaf fiber (PALF) and roselle fiber is expected to produce a synergistic effect due to their complementary properties. PALF, known for its high tensile strength and stiffness, enhances load-bearing capacity, while roselle fiber contributes to improved impact resistance and toughness. The combination also promotes better stress distribution and crack arresting mechanisms^26,27^. Furthermore, the distinct surface textures of the two fibers can enhance interfacial bonding with the epoxy matrix. This synergy is anticipated to result in superior mechanical performance compared to single-fiber reinforced composites.

Muthuraja et al.^26^ investigated the mechanical properties and tribological behavior of PALF and roselle fiber reinforced with vinyl ester composites. The work summarized that a hybrid combination of 24 wt% PALF and 16 wt% roselle attains superior mechanical properties and minimizes wear. Glória et al.^28^ studied the tensile effects of polyester composites reinforced with PALF and found that significant tensile results depend on the amount of PALF fiber. Jothiprakash et al.^27^ worked on a hybrid combination of PALF and kenaf fiber reinforced vinyl ester composites and evaluated their mechanical and tribological behavior. The results report that the 20PALF/20KF/60VE sample has higher mechanical properties and is suggested for switchboard and printed circuit board applications.

### Tribological importance

The mechanical and wear performance of natural fiber composites is closely linked to the fiber–matrix interaction, which depends on the fiber’s chemical composition and geometry. PALF has high cellulose content and a high aspect ratio, contributing to excellent tensile strength and efficient stress transfer^29^. Roselle fiber, with moderate lignin content and good flexibility, enhances toughness and impact resistance. The combined presence of these fibers promotes better mechanical interlocking and load distribution within the epoxy matrix. Such structure property relationships are key to achieving improved overall composite performance^30^. Vikas Yadav et al.^31^ studied the sliding wear behaviour of laminated composites by incorporating sisal, bagasse, and banana fibers with poly-lactic acid by injection moulding in 10% and 20% fiber concentrations. The wear test was performed by pin-on-disc tribometer and suggests that specific wear rate and frictional coefficient was reduced with improve in fiber content and through the chemical treatment favoured in enhancing the tribological properties. Somen Biswal et al.^32^ explores the wear characteristics of epoxy composite reinforced with short Palmyra fibers with different proportions of 0, 4, 8, and 12 wt% by hand lay-up method. From the L16 experimental analysis, the properties of wear resistance of the composite were improved with increased fiber content. The most influential parameter that affects the wear performance were fiber content under by velocity of sliding, distance, and applied weight. Irullappasamy Sankar and Durairaj Ravindran^33^ focussed on the dry sliding wear behaviour and mechanical properties of Palmyra fruit fiber reinforced polyester composites. Based on the NaOH alkali treatment, the mechanical interlocking between the fiber and resin improves the mechanical properties of the composite. The surface modified composite sample by alkali treatment, wear resistance also enhanced with increased applied load sliding velocity. Banu Murali et al.^34^ explored the wear behaviour of hybrid epoxy composite reinforced with bamboo, Kevlar, aloe vera, and palm fibers by vacuum-assisted compression moulding process. Based on the RSM and GRA approaches, the ideal combination for better wear resistance were 5 N applied load, 3 m/s sliding velocity, and 1500 m sliding distance. Micro-striation pattern and fiber delamination were majorly identified as wear mechanisms during sliding. Compared to previously reported PALF/kenaf hybrid composites, the PALF and roselle fiber composites in superior wear resistance. This improvement was attributed to the higher cellulose content and stiffer nature of PALF, combined with the relatively tougher and more flexible roselle fiber, which together reduce surface damage during sliding. Additionally, the improved fiber–matrix adhesion observed from SEM images further supports the enhanced wear performance. These findings suggest that the PALF/roselle combination offers a balanced reinforcement, outperforming some existing natural fiber hybrid systems in wear applications^35^.

### Literature gap and novelty

Several studies have explored natural fiber reinforced composites, the hybridization of pineapple leaf fiber and roselle fiber remains largely unexplored. Also, previous works that primarily used hand lay-up molding, this study employs hot compression molding to ensure better fiber wetting and void reduction, resulting in improved mechanical properties. The prime novelty of the work is hybridizing PALF with roselle fibers and reinforcing them with epoxy resin, processed by hot compression molding, and no research study has been conducted and published in this combination. Moreover, the mechanical properties of the composites, such as tensile, flexural, and impact tests along with dry sliding wear behaviour were evaluated. The respective fractography and worn surface of the specimen were analyzed through SEM to endorse the optimized composite sample for commercial cabinet applications like wardrobes, cupboards, and storage shelves.

## Methods and materials

### Base materials

The raw fibers of PALF and roselle are collected from the regional zone of the southern agricultural regions of Tiruchirappalli, India. The collected fibers were manually extracted from raw pineapple leaves and roselle stems through retting and mechanical scraping, followed by washing and drying to remove impurities. The commercially available matrix, catalyst, and promoter, namely epoxy resin, methyl ethyl ketone peroxide, and N, N dimethylaniline, are procured from Covai Seenu Enterprises, Coimbatore, India. The general properties of the employed fibers were presented in Table 1. The epoxy resin was mixed with a hardener in a 10:1 weight ratio, and a promoter (cobalt naphthenate) and catalyst (methyl ethyl ketone peroxide) was added at 1.5% and 1% by weight of the resin to initiate the curing process. The selection of PALF and roselle fiber is based on their favorable mechanical properties, such as high tensile strength, moderate elongation, and good aspect ratio, as shown in Table 1. These characteristics make them technically suitable for reinforcing polymer matrices, offering potential improvements in strength, stiffness, and impact resistance while maintaining a lightweight and sustainable profile.

**Table 1:** General properties of roselle and pineapple leaf fiber.

Properties	Roselle fiber	Pineapple leaf fiber
Density (g/cc)	1.49	1.44
Hemicellulose (%)	31–38	18
Cellulose (%)	48.5–56.6	70–82
Lignin (%)	11.6–12.5	5–12
Tensile strength (MPa)	135–159	413–1627
Elongation at break (%)	0.4–0.6	1.6
Young’s modulus (MPa)	19–23	34–82

### Alkaline treatment

The fibers were rinsed thoroughly with distilled water and then sun-dried for a cumulative duration of 45 h over a period of 7 days to ensure complete removal of moisture. The dried fibers are soaked in diluted sodium hydroxide (5 wt%) with distilled water for 1.5 h. This surface modification technique notably enhances fiber-matrix adhesion and the overall performance of fibre composites^36^. Then, the soaked fibers are washed with fresh water to remove the NaOH presence and dried out in an air-oven for 12 h. The long fibers are chopped into small fibers with an average size of 20 ± 2 cm.

### Preparation of composites

The weighted chopped fibers of PALF and roselle are arranged randomly between the two stainless steel plates and compressed by a 30-ton hydraulic compression machine. The compressed randomly oriented fiber mats are placed in a mold with dimensions of 300 × 300 × 3 mm. The proper proportions of epoxy resin mixed with catalyst, hardener, and promoter are poured over the fiber mat arrangements. Then, it was sealed by stainless steel plates, and using a hot compression molding machine, composite plates were prepared by applying a pressure of 2.5 MPa for 50 min at a molding temperature of 100 °C.

The prepared composite sample is unmounted from the arrangements and allowed to cure for 24 h at room temperature (28 ± 2 °C). By following similar fabrication steps, the designated forms of samples (Table 2) are fabricated and prepared for mechanical tests using a conventional electrical hacksaw machine.

**Table 2:** Composite details and weight fraction.

Composite sample specification	Designation of composites	Weight fraction (wt%)	
		PALF	Roselle
S1	0PALF/0RF/100E	0	0
S2	0PALF/50RF/50E	0	50
S3	50PALF/0RF/50E	50	0
S4	20PALF/30RF/50E	20	30
S5	25PALF/25RF/50E	25	25
S6	30PALF/20RF/50E	30	20

### Mechanical and morphological characterization

The mechanical characteristics, like tensile and flexural properties are performed on the composite samples using a universal testing machine (Tinius-Olsen H-10 KL) with a crosshead speed of 1 mm/min as per ASTM D638 and ASTM D790. The impact testing was executed as per ASTM D256 using a computerized pendulum-type Izod impact tester (Tinus-Olsen Impact 104). The mechanical tests were conducted on three specimens per sample, and the average was noted for analysis including with error bar (mean ± standard deviation) for consistent results for discussion. The fractured surfaces of the tensile and impact specimens and worn surfaces were examined using SEM (ZEISS Sigma 300).

### Dry sliding wear test

The sliding wear behaviour of the composite was tested by pin-on-disc tribometer (DUCOM TR-20) and the photographic view of the pin-on-disc wear test setup used to analyze the dry sliding wear behaviour of the composites is shown in 2. The composite samples of dimension ϕ 30 × 10 mm were prepared as pins by following the standard ASTM G99-05 for testing against the EN 31 hardened steel disc material with the hardness level of 62 ± 2 HRC (Rockwell hardness – C). The pins were rubbed with the abrasive paper of grit size 1000 and 1200, to maintain the initial test condition. Also, the disc face was also scrubbed with the abrasive papers and wiped with acetone to retain the ideal condition after each test.

The wear test was conducted under constant time period of 30 min at a speed of 1.5 m/s by applying the load of 15 N in the ambient temperature condition (24 ± 2 °C), respectively accordingly by following former studies^28^. After sliding, the surface of the tested pin was thoroughly cleaned with acetone-soaked tissue paper followed by drying in hot air. This difference between the starting weight of the pin being tested and its end weight is what is used to calculate the sliding wear loss. The prepared pin was primarily weighted in an electronic weighing balance with an accuracy of 0.0001 g. Two number of tests were taken from each sample on the top and bottom areas and their average was used for the analysis. For the purpose of measuring sliding wear, the weight loss of the pin was used, and the specific wear rate was determined by employing Eq. (1)^26^.

1 $$Specific~wear~rate~\left( {{W_s}} \right)=~\frac{{\Delta w}}{{\rho D{F_n}}}$$

Where ‘$\Delta w$’ is the weight loss in ‘gm’, ‘$\rho$’ is the density of the test specimen in ‘gm/cc’, ‘$D$’ is the sliding distance in ‘m’, and ‘${F_n}$’ is the applied load in ‘N’.

During the beginning of the sliding test between the pin and the disc, the wear and friction monitor displayed the frictional force (F_f_) in a continuous manner. It was also noticed that the coefficient of friction could be computed by utilizing Eq. (2)^26^.

2 $${\text{Coefficient~of~friction~}}\left( \mu \right)=~\frac{{Frictional~force~\left( {{F_f}} \right)}}{{Applied~load~\left( {{F_n}} \right)}}$$

Where $\:{\prime\:}{F}_{f}{\prime\:}$ is the frictional force and $\:{{\prime\:}F}_{n}{\prime\:}$ is the applied load

## Results and discussion

### Tensile strength evaluation

**Fig. 1:**
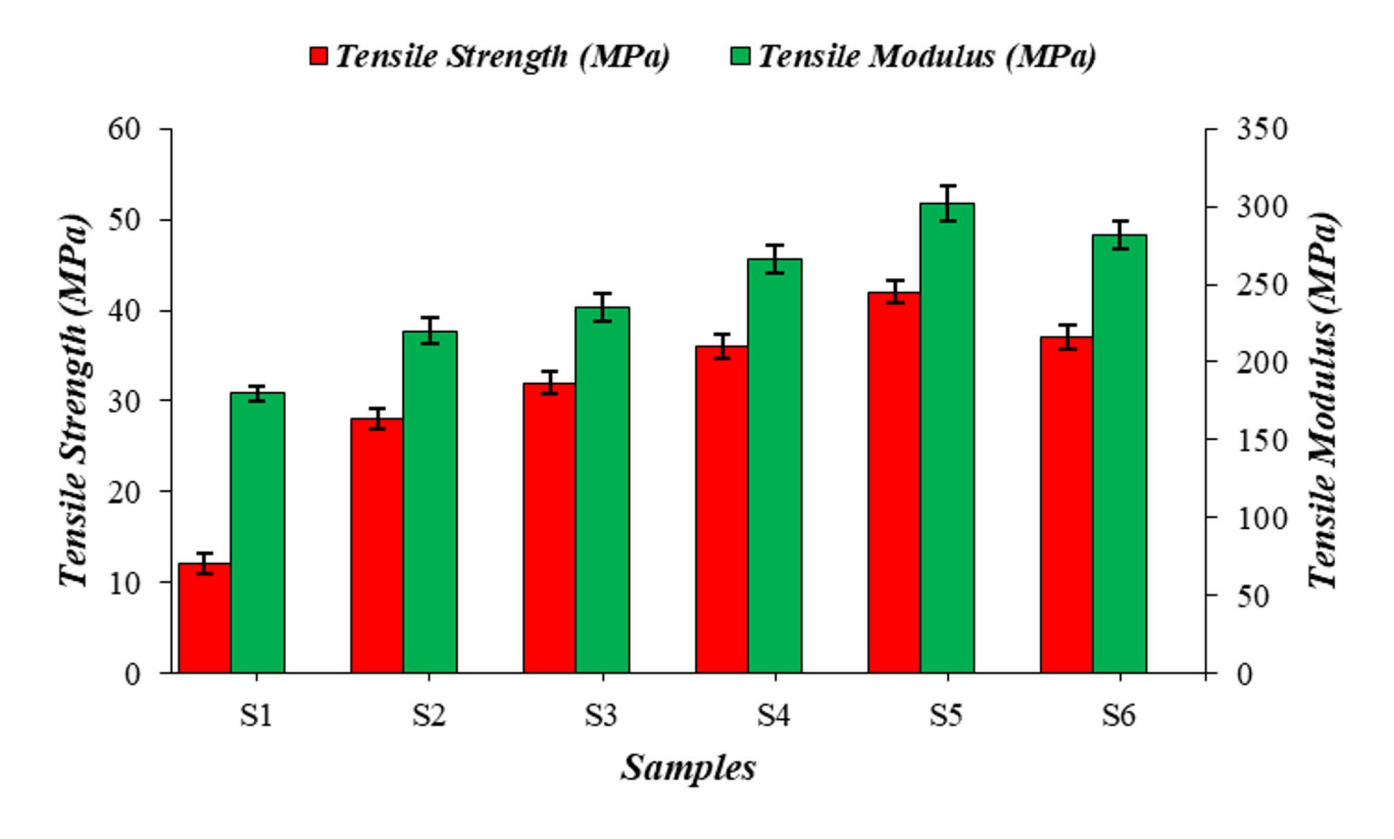
Graphical representation on tensile strength of the composite samples.

The tensile test result of the composites is shown in Fig. 1, from the mean of three specimens from each sample. The tensile strength of the neat resin sample (S1) achieved was 12 MPa. The addition of PALF and roselle fibers subsequently increases the tensile strength of the samples. The single-fiber composite samples (S2 & S3) even have higher tensile results than those of the S1 sample. The fibers possess significant mechanical aspects like high tensile strength and modulus. This factor contributes to developing the load bearing capacity of the composite samples^37^. Among the hybrid reinforced samples, S5 (25PALF/25RF/50E) has attained a maximum strength of 42 MPa. This enhancement was mainly due to the strong adhesion and improved stress transfer between fibers and matrix^38^. The combination of PALF and roselle generates a synergistic effect and good adhesion between the matrixes. These act as primary load carrier and reduces the stress concentration in the matrix and allows the composite to withstand higher tensile load^39^. In the case of S6 (37 MPa), the uneven distribution of fibers limited the strength of the composites. This dispersion in the matrix generates weak points and reduce the effectiveness of the fibers. Compared to single-fiber composites (S2 and S3), hybrid composites (S4, S5, and S6) attained higher tensile values, because the fiber-to-fiber interaction was greater and improved mechanical interlocking. Therefore, the presence of hybrid fibers increases energy absorption during stress and delaying the fracture propagation^40^. This results in increased tensile strength. Similar to the trend of tensile strength, tensile modulus is improved due to the stiff nature of the fibers which restricts the deformation of the composites under tensile loads.

#### Tensile fractography analysis

**Fig. 2:**
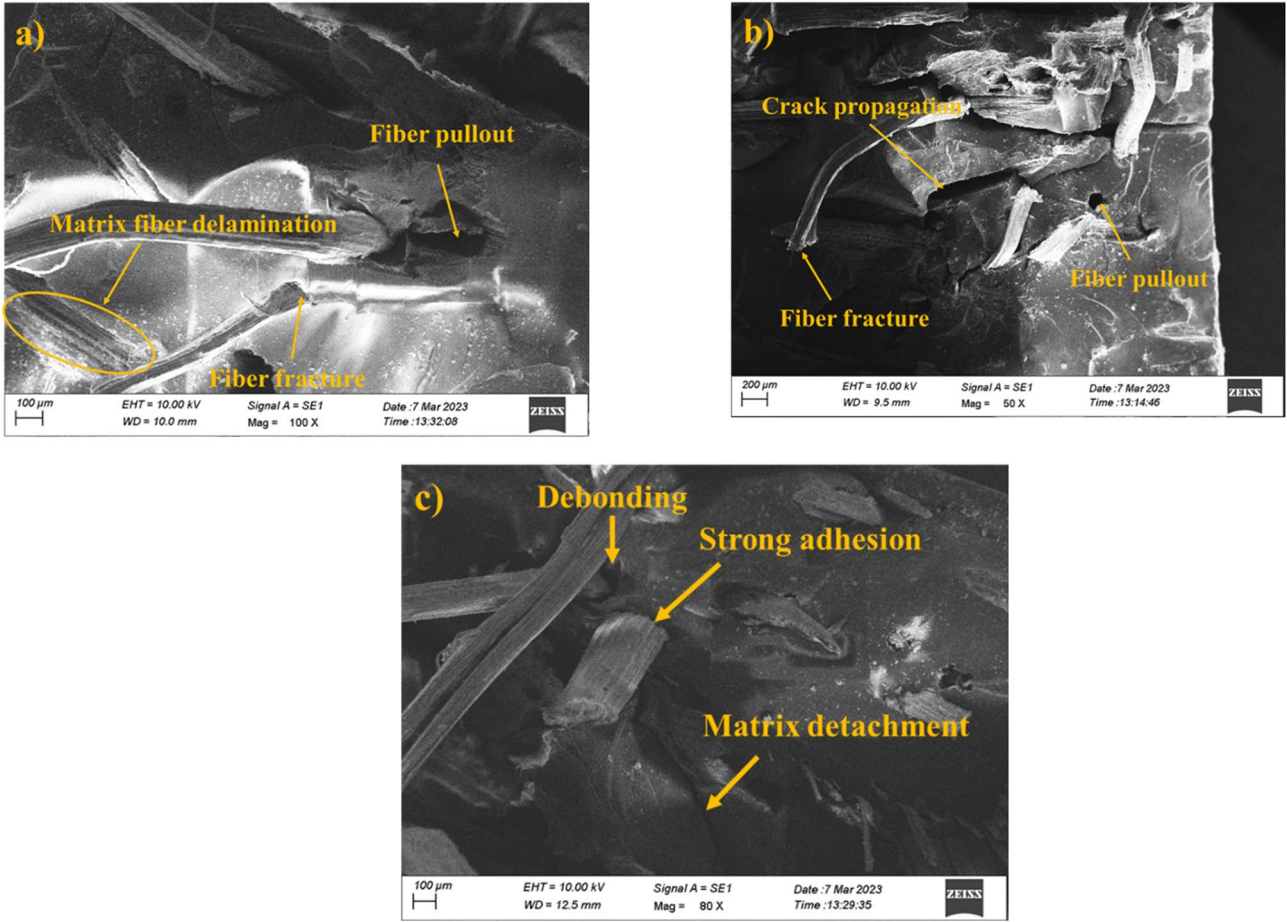
Fractography images of tensile fractured samples (a) 20PALF/30RF/50E (S4), (b) 25PALF/25RF/50E (S5), and (c) 30PALF/20RF/50E (S6).

The fractured surfaces of the hybrid composite samples were analysed through SEM shown in Fig. 2 (a-c) and their effects were revealed. The improved tensile strength observed aligns with findings from cellulose-reinforced PLA biocomposites^41^, where extracted natural fibers enhanced polymer matrix performance. The fractography analysis of the hybrid composite sample revealed a range of failure mechanisms like fiber debonding, fiber pullout, matrix delamination, crack propagation, fiber debonding, strong adhesion, and matrix detachment. Fiber pullout observed on the fracture surfaces (Fig. 2a and b) specifies a fractional loss of interfacial bonding between the fibers and the matrix. This failure approach suggests that the adhesion was insufficient in some regions to completely transfer the tensile load to the fibers and leads to the fibers pulled out rather than fractured. Matrix-fiber delamination was another significant failure mechanism caused due to the weak interfacial adhesion at the fiber-matrix interface shown in Fig. 2c. This which finally unite into larger cracks and accelerate the failure process^42,43^.

The fractography test were observed for S4, S5, and S6 samples, because S4 exhibit improved mechanical strength, S5 with enhanced mechanical and wear beahviour, and S6 with better wear properties. Fiber fracture was observed from Fig. 2a–c along with evidence of crack propagation, reflects the load-bearing factor of the fibers in resisting tensile stress. The fractured fibers which indicate the load was effectively transferred from the matrix to the fibers in certain regions and signifies the formation of strong adhesion at the fiber-matrix interface^44^. Crack propagation patterns shown in Fig. 2b, exposed that crack initiated at weak spots. Moreover, strong adhesion was observed in some regions (Fig. 2c) indicates optimized bonding between the fibers and matrix which cohesively resist the tensile stresses and delaying the commencement of failure^45^. Overall, the tensile fractographic results reveal the critical role of the interface in analysing the tensile performance of hybrid composites (S4, S5, and S6).

### Flexural test analysis

**Fig. 3:**
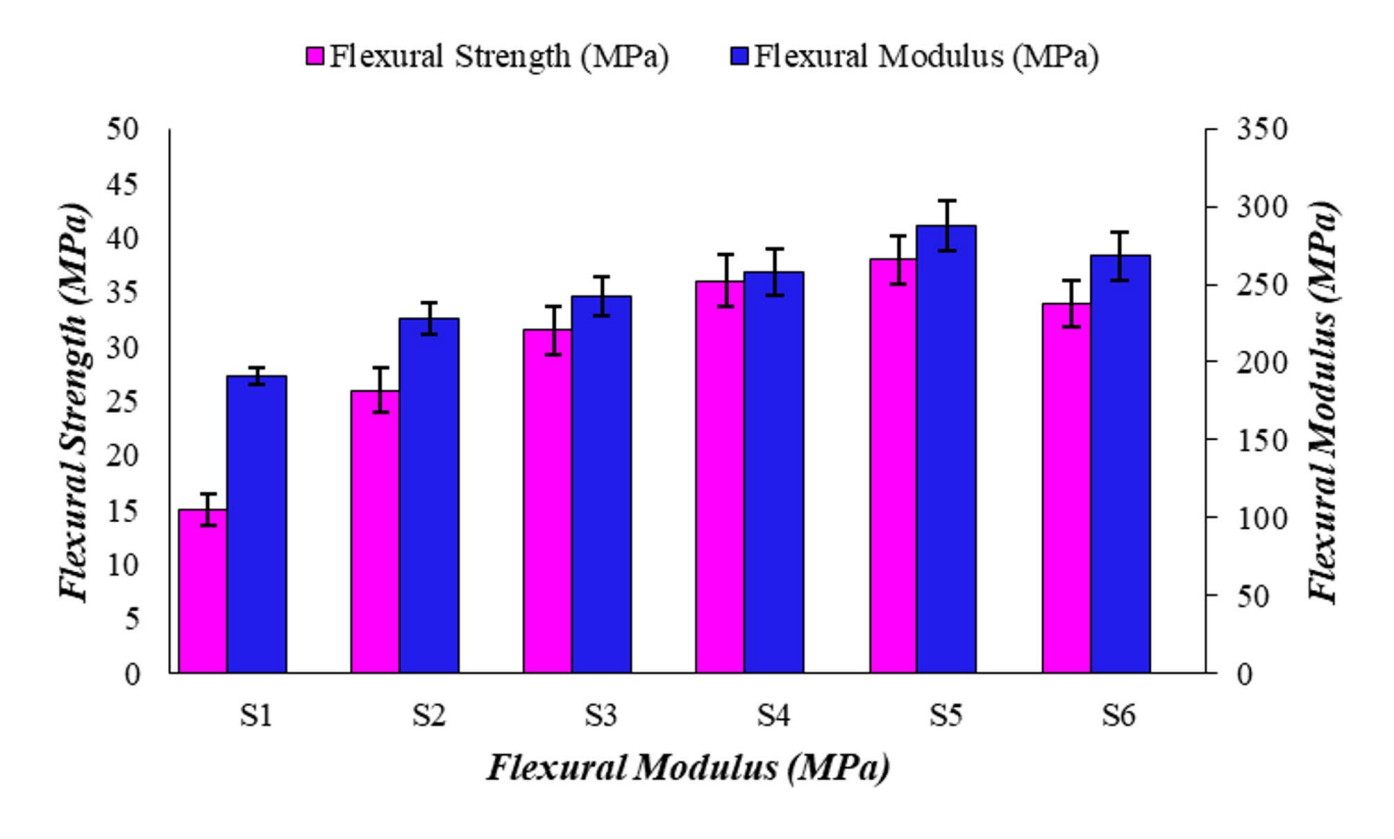
Graphical analysis on Flexural strength of the composite samples.

A three-point bending test was conducted, and the respective results are shown in Fig. 3. Analyzing the results, hybrid-fiber reinforced samples (S4, S5, and S6) had higher flexural strength than the single-fiber composite (S2 and S3) and neat resin samples. The influence of natural fibre on improving matrix properties has been evidenced in vinyl ester systems^46^, and comparable advantages are noted in the epoxy-based PALF/roselle composites presented here. The addition of fibers allows for better distribution of flexural stress and less prone to cracking under bending. The peak result attained for the S5 (25PALF/25RF/50E) sample was 46 MPa, while the least for the neat resin sample (S1) was 22 MPa. During flexural testing, the upper surface, the upper surface area of the composite experiences a compression stress and the lower region is under tension. The observed synergistic effect in hybrid composites mirrors results from nanomaterial-reinforced polymers^47^, where filler dispersion critically influenced mechanical properties. This stress balance is enhanced by hybrid fibers with dissimilar stiffness and elongation properties, improving overall flexural strength and modulus^38,39^.” Also, the proper blending action and strong interface strength were attained between fibers and matrix, resulting in improved flexural strength and modulus.

The hybridization of PALF and roselle fibers restricts the crack propagation in the matrix and improves the hybrid composite ability to withstand higher flexural stress and modulus^40^. Moreover, during the usage of hybrid fiber, one fiber can bridge weak zones left by the other fiber and ensures the resistance to bending stresses. Similar to the tensile analysis, single-fiber composites have a limited strength value because of the presence of only PALF or roselle fiber, which does not provide enough cross-linking density between fibers and matrix^28^. The variation in stiffness between PALF and roselle fibers influenced the internal stress distribution within the composite. The stiffer PALF fibers provided higher load-bearing capacity, while the relatively more flexible roselle fibers contributed to energy dissipation and crack deflection^35^. This mismatch in stiffness generated a synergistic effect, leading to improved mechanical strength and controlled failure mechanisms, as evidenced in the flexural analysis^29,30^.

### Impact test analysis

**Fig. 4:**
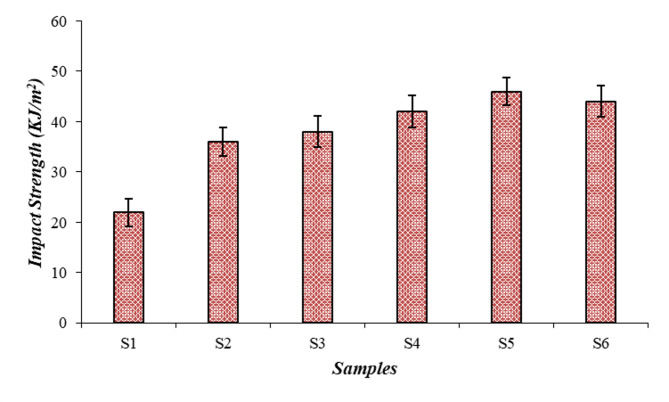
Graphical view on impact strength of the composite samples.

The Izod test was performed to analyze the impact strength of the composite samples, and the results are shown in Fig. 4. The result implies that the S5 (25PALF/25RE/50E) sample achieved a higher impact result of 46 KJ/m^2^, which was 109% higher than the S1 sample. The impact test also follows the same trend as the tensile and flexural tests. The higher cellulose content of the fiber enhances the binding action and crack-bridging mechanism between the fibers and the matrix, resulting in improved impact strength^26^. The roselle fibers absorb impact energy successfully due to their toughness and PALF fibers enhance structural rigidity, leads to superior impact resistance of composite. The inclusion of fiber in the matrix enhances the composite ability to absorb and dissipate kinetic energy imparted during the impact. When the PALF wt% increased (S6), the result was reduced to 44 KJ/m^2^.

The higher rigidity of PALF can result in more brittle behavior, and the difference in wt% of fibers also affects the impact performance of the composite^28^. The synergistic effect of PALF and roselle fibers serves as crack stoppers and effectively arrest the propagation of cracks originated by impact and delays the catastrophic failure and increases the energy required to fracture^38,39^. From the results, the S6 sample with the increased weight of PALF fibers (30 wt%) leads to a higher rigidity and limits the composite ability to deform plastically under dynamic loads which results in a more brittle response to impact^40^. Thus, the improvement in impact strength for single-fiber (S2 and S3) and hybrid composites (S4, S5, and S6) is determined by synergistic fiber interactions, energy dissipation, and crack-arresting mechanisms.

#### Impact fractography analysis

**Fig. 5:**
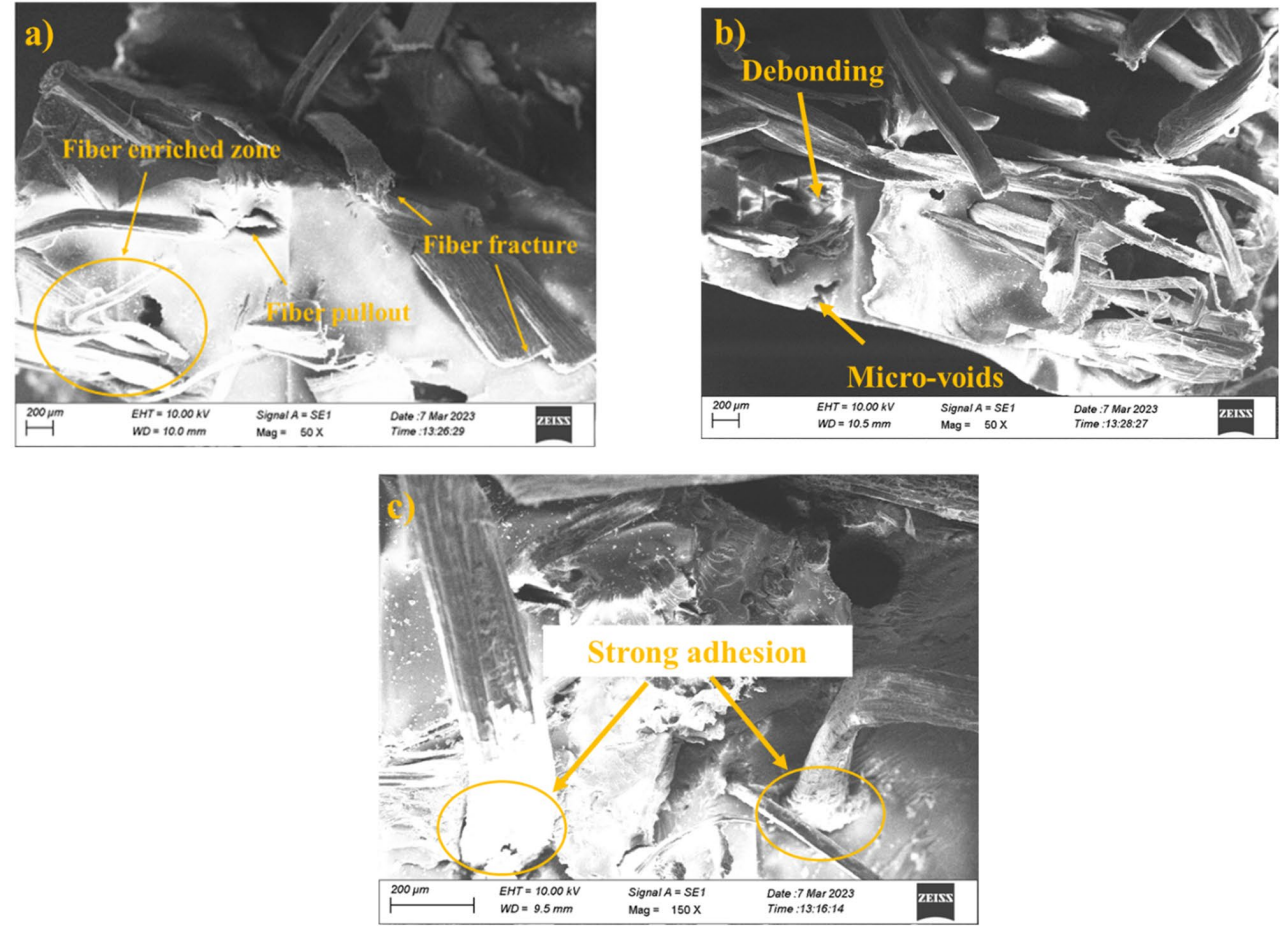
Fractography images of tensile fractured samples (a) 20PALF/30RF/50E (S4), (b) 25PALF/25RF/50E (S5), and (c) 30PALF/20RF/50E (S6).

The impact tested hybrid composite specimen (S4, S5, and S6) fractured surfaces were analysed and their results were shown in Fig. 5a–c. The impact fractography analysis of the hybrid fiber composites revealed critical insights into their energy absorption and failure mechanisms under dynamic loading. From the Figures, the features such as fiber-enriched zones, fiber fracture, fiber pullout, debonding, microvoids, and strong adhesion were observed on the fracture surfaces.

The SEM image Fig. 5a shows densely packed fiber regions with minimal voids, indicating strong fiber–matrix adhesion. These fiber-enriched zones facilitated efficient load transfer and crack deflection, thereby contributing significantly in absorbing the impact energy. These zones suggest that the PALF and roselle effectively reinforced and acts as primary load carriers and dissipating the energy through mechanisms such as fiber deformation, fractures, and pullout^48,49^.

Fiber fracture (Fig. 5a) was prominently observed and signifying that the fibers bore a substantial portion of the impact load until the specimen failed. This failure highlights the importance of fiber strength and their contribution to the hybrid composite impact toughness. Another key observation found was fiber pullout (Fig. 5a), indicates regions where the interfacial bond strength between the fibers and matrix was insufficient under the high strain rates of impact loading^42,43^. The fiber pullout mechanism is energy intensive and contributes to the hybrid composite energy absorption. Debonding was observed at the fiber-matrix interface (Fig. 5b) suggests that localized stress concentrations caused interfacial failure during the impact. Also, microvoids seen from the results are often formed due to stress-induced cavitation and act as stress concentrators. It accelerates crack initiation, growth, and reducing the hybrid composite overall toughness^44^. Finally, a well-bonded fiber surfaces were observed from Fig. 5c that fibers and matrix resists separation and contributes to delayed crack growth and higher energy absorption^45^. Thus, the fractographic features of the impact tested hybrid composite specimen highlights the importance of fiber distribution, adhesion, and matrix toughness to attain improved impact resistance.

### Sliding wear behaviour

#### Specific wear rate

**Fig. 6:**
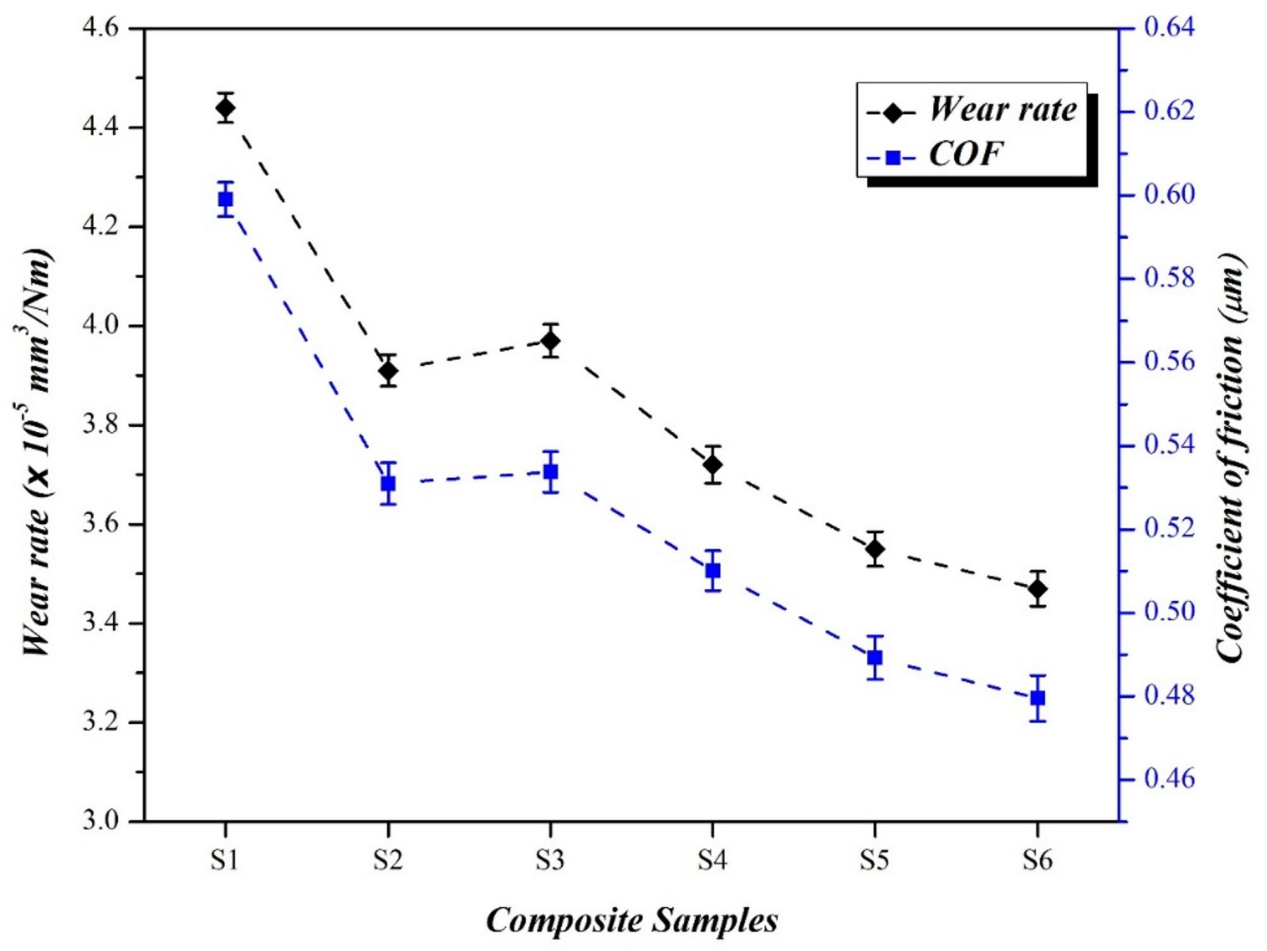
Wear behaviour results of the composite samples.

The specific wear rate is the significant property for evaluating the tribological behaviour of the fabricated composites and the testing results are shown in Fig. 6. The neat resin (S1) sample exhibits the highest wear rate of 4.31 × 10^− 5^ mm^3^/Nm and expresses their unsuitability to the wear conditions. These results also highlight the necessity of reinforcement to enhance the wear resistance. Due to the inherent strength and stiffness of the reinforcement, the composite samples (S2 – S6) effectively improve the wear resistance and indicating that PALF and roselle fibers acts as a barrier to material degradation during sliding action. Compared to single fiber composites (S2 and S3), hybrid reinforcement with different weight combinations of PALF and roselle achieved a lowest specific wear rate. This is because the good tensile strength nature of PALF combined with high stiffness roselle fiber likely reduced the material loss during sliding^26,31^. The wear rate was reduced to 3.52 × 10^− 5^ mm^3^/Nm for S5 sample and indicates that an optimal balance between the fiber contributions provide resistance to abrasive forces and increasing the load bearing capacity of the composite^32^. The finest wear behaviour was achieved for the sample with 30 wt% PALF and 20 wt% roselle (S6) sample of 3.45 × 10^− 5^ mm^3^/Nm specific wear rate. The composite sample exhibited a 19.95% improvement in wear resistance compared to the neat resin sample (S1), indicating the effectiveness of fiber reinforcement in reducing material loss during sliding.

The improved interfacial bonding generated between the fibers and epoxy matrix promotes energy absorption and reduces the material loss during sliding and results in enhanced wear resistance^33^. These findings illustrate that hybrid reinforcement of PALF and roselle with epoxy matrix significantly improves wear resistance properties and potential for practical applications.

#### Coefficient of friction

In this study, the coefficient of friction values for composite samples with varying weight contributions of PALF and roselle fiber with epoxy matrix were determined and the results are shown in Fig. 6. The COF result attained was like the trend of specific wear results. This behaviour is attributed to the frictional force generated at the contact interface during the sliding experiment, which influenced the material removal mechanism and wear rate. The sample without reinforcement (S1) sample getting the maximum COF results of 0.599 μm and reflecting the inherent frictional properties of the epoxy matrix. The maximum improvement of COF was achieved for the sample (S6) with 30 wt% PALF and 20 wt% roselle fiber getting 0.480 μm and representing a 19.86% reduction compared to neat resin (S1) sample.

Similarly, the hybrid reinforcement of PALF and roselle fiber with epoxy matrix (S4, S5, and S6) getting reduced COF than the single fiber composites (S2 and S3) due to the synergistic effect of the fibers. The roselle and PALF contribute to the composite ability to bear loads and minimize material loss during sliding action. The primary reason for the improvement in the coefficient of friction is the inherent mechanical properties of the PALF and roselle fiber reinforcement. These fibers, when integrated with the epoxy matrix, acts as reinforcing agents and distributing the load evenly across the surface and reducing localized stress concentration^31–33^. These factors reduce material deformation and contact area during sliding, resulting in a lower coefficient of friction (COF). Another significant factor that enhances the wear resistance is provided by the fibers. The fibers provide the barrier effect during sliding and improve the durability of the composite sliding surface. The fibers act as micro-lubricants and reducing adhesion between the contacting surfaces and results in reduced COF. Also, the thermal stability of the fibers reducing the thermal softening of the epoxy matrix and maintains the structural integrity during sliding^34^. These combined actions make the significant improvement in COF of hybrid fiber reinforced composite samples (S4, S5, and S6).

#### Worn surface analysis

**Fig. 7:**
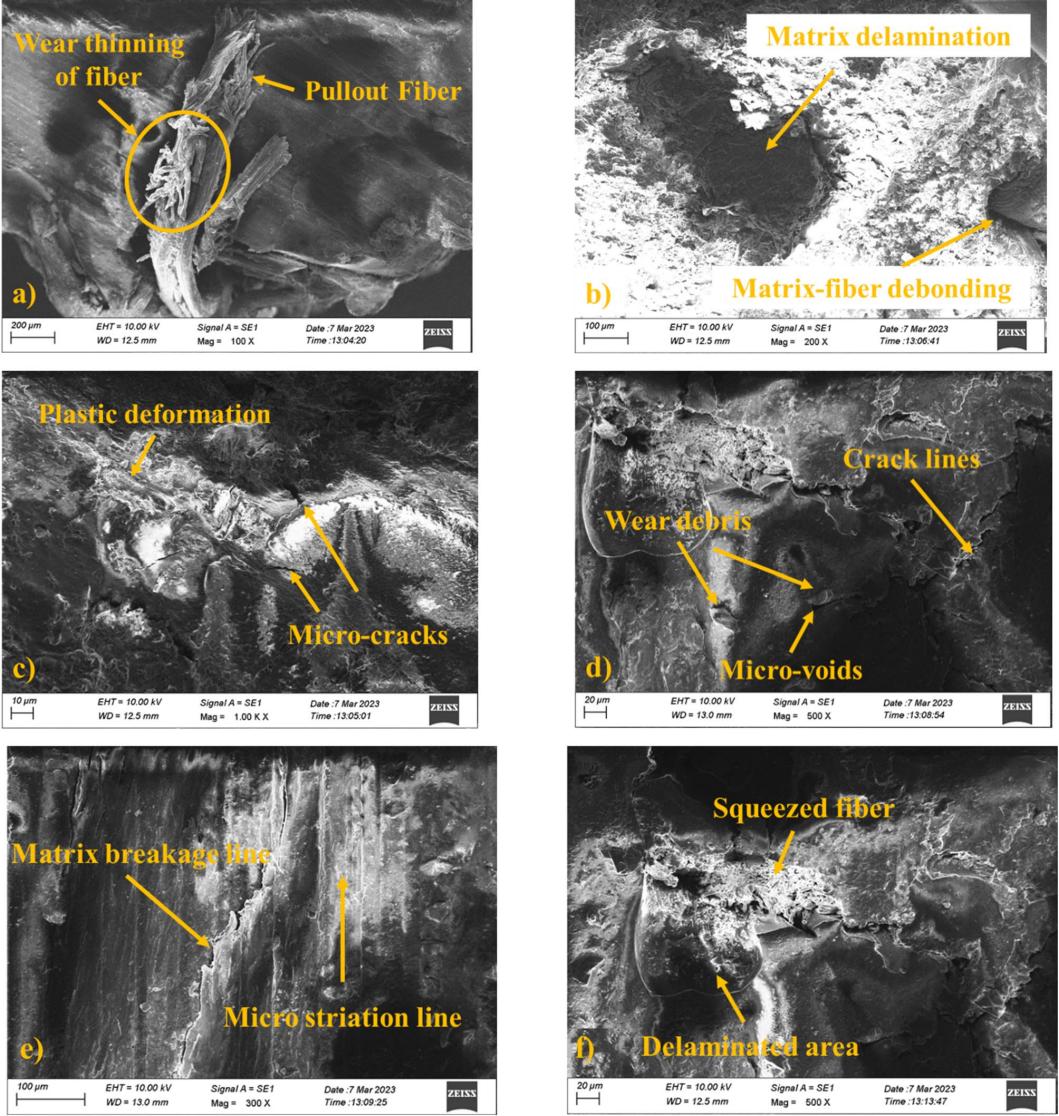
Worn surface analysis of composite fractured samples (a-b) 20PALF/30RF/50E (S4), (c-d) 25PALF/25RF/50E (S5), and (e-f) 30PALF/20RF/50E (S6).

The worn surface of the composite specimen (S4, S5, and S6) was investigated through SEM and the results are shown in Fig. 7a–c. The worn surface analysis of the composites revealed various wear mechanisms and defects such as fiber thinning, fiber pullout, matrix-fiber debonding, matrix delamination, and plastic deformation. These defects indicate the complex forces acts during the sliding process. Fiber thinning was observed on the worn surface of sample 4, suggests that the fibers reinforced absorbs major amount of frictional energy and enhancing the complete wear resistance. But the presence of fiber pullout (Fig. 7a) points that insufficient interfacial bonding between the fibers and the matrix in certain regions. This leads to limited material removal and increased friction^50^. From Fig. 7b, matrix-fiber debonding features the role of proper adhesion at the fiber-matrix interface and ensuring the effective load transfer. Matrix delamination and plastic deformation was observed from Fig. 7b on the hybrid composites indicate the matrix role in accommodating the wear induced stresses. Plastic deformation of the matrix was observed from Fig. 7c, suggest that the matrix undergoes a ductile behavior under sliding stress and prevents the brittle failure. This led to surface irregularities and influence the coefficient of friction^51^.

Microcracks and microvoids from Fig. 7d, e indicate fatigue failure and material degradation during the wear and contributes to the detachment of matrix and fiber fragments as wear debris. The wear debris generation was further worsening surface abrasion and forming secondary wear pathways and affects the hybrid composite long-term performance^52,53^.

Other surface features observed from the worn surfaces of sample 6 (Fig. 7e and f) are micro-striation lines, squeezed fibers, and delaminated areas. Micro-striation lines indicate the repeated frictional contact and abrasion, which result in fatigue induced damage^54^. Squeezed fibers observed on the surfaces highlight the load-bearing ability of the fibers during sliding^55^. Finally, the presence of delaminated areas reveals the localized failures in fiber reinforcement. These findings underscore the challenges related with hybrid fiber composites and discussed the significance of achieving strong interfacial bonding and uniform fiber distribution to enhance tribological performance with minimized wear defects.

## Conclusion

The pineapple leaf fiber and roselle fiber reinforced samples were successfully fabricated, and their mechanical properties were investigated. From the results, the following conclusions are drawn:

- Overall analysis reveals that compared to single-fiber composites; hybrid-fiber composite samples achieve better mechanical properties.
- The tensile test result indicates that the 25PALF/25RF/50E (S5) sample has a higher tensile strength of 42 MPa and moduli of 302 MPa. It was comparatively 250% and 68% improved over the neat resin (S1) sample.
- Along with flexural and impact results, it also implies that the 25PALF/25RF/50E (S5) sample attains a peak flexural value of 38 MPa and an impact result of 46KJ/m^2^. The equal weight fraction of the hybrid fibers increased the performance of the results.
- Fractographic analysis of the tensile, and impact samples noted the presence of failure mechanisms like fiber debonding, fiber pullout, fiber fracture matrix-fiber delamination, crack propagation, fiber debonding, strong adhesion, and matrix detachment.
- Lowest specific wear rate and co-efficient of friction attained was 3.45 × 10^− 5^ mm^3^/Nm and 0.480 µ for 30PALF/25RF/50E (S6) hybrid composite sample.
- The worn surface analysis revealed wear mechanisms and defects such as fiber thinning, fiber pullout, matrix-fiber deboning, matrix delamination, and plastic deformation.

From the results, it was suggested that a hybridized form of PALF and roselle fiber reinforced epoxy composites is the ideal material combination to support commercial cabinet applications like wardrobes, cupboards, and storage shelves. Also, the present work was limit to mechanical behaviour analysis. The future research could focus on thermal property analysis and water absorption behavior to better understand the long-term durability of the hybrid composites.

## Data Availability

The data used to support the findings of this study are included within the article.
